# Investigate on the Mechanism of HfO_2_/Si_0.7_Ge_0.3_ Interface Passivation Based on Low-Temperature Ozone Oxidation and Si-Cap Methods

**DOI:** 10.3390/nano11040955

**Published:** 2021-04-09

**Authors:** Qide Yao, Xueli Ma, Hanxiang Wang, Yanrong Wang, Guilei Wang, Jing Zhang, Wenkai Liu, Xiaolei Wang, Jiang Yan, Yongliang Li, Wenwu Wang

**Affiliations:** 1 School of Information Science and Technology, North China University of Technology, Beijing 100144, China; yaoqide@ime.ac.cn (Q.Y.); wanghanxiang@ime.ac.cn (H.W.); zhangj@ncut.edu.cn (J.Z.); liuwk@ncut.edu.cn (W.L.); yanjiang@ncut.edu.cn (J.Y.); 2 Integrated Circuit Advanced Process Center, Institute of Microelectronics, Chinese Academy of Science, Beijing 100029, China; wangguilei@ime.ac.cn (G.W.); wangxiaolei@ime.ac.cn (X.W.); wangwenwu@ime.ac.cn (W.W.)

**Keywords:** HfO_2_/Si_0.7_Ge_0.3_ gate stack, ozone oxidation, Si-cap, interface state density, passivation

## Abstract

The interface passivation of the HfO_2_/Si_0.7_Ge_0.3_ stack is systematically investigated based on low-temperature ozone oxidation and Si-cap methods. Compared with the Al_2_O_3_/Si_0.7_Ge_0.3_ stack, the dispersive feature and interface state density (D_it_) of the HfO_2_/Si_0.7_Ge_0.3_ stack MOS (Metal-Oxide-Semiconductor) capacitor under ozone direct oxidation (pre-O sample) increases obviously. This is because the tiny amounts of GeO_x_ in the formed interlayer (IL) oxide layer are more likely to diffuse into HfO_2_ and cause the HfO_2_/Si_0.7_Ge_0.3_ interface to deteriorate. Moreover, a post-HfO_2_-deposition (post-O) ozone indirect oxidation is proposed for the HfO_2_/Si_0.7_Ge_0.3_ stack; it is found that compared with pre-O sample, the D_it_ of the post-O sample decreases by about 50% due to less GeO_x_ available in the IL layer. This is because the amount of oxygen atoms reaching the interface of HfO_2_/Si_0.7_Ge_0.3_ decreases and the thickness of IL in the post-O sample also decreases. To further reduce the D_it_ of the HfO_2_/Si_0.7_Ge_0.3_ interface, a Si-cap passivation with the optimal thickness of 1 nm is developed and an excellent HfO_2_/Si_0.7_Ge_0.3_ interface with D_it_ of 1.53 × 10^11^ eV^−1^cm^−2^ @ E−E_v_ = 0.36 eV is attained. After detailed analysis of the chemical structure of the HfO_2_/IL/Si-cap/Si_0.7_Ge_0.3_ using X-ray photoelectron spectroscopy (XPS), it is confirmed that the excellent HfO_2_/Si_0.7_Ge_0.3_ interface is realized by preventing the formation of Hf-silicate/Hf-germanate and Si oxide originating from the reaction between HfO_2_ and Si_0.7_Ge_0.3_ substrate.

## 1. Introduction

High-mobility channel materials and novel device architectures, such as FinFETs (Fin Field-Effect Transistor) and nanowire FETs, are proposed to address the demand for scaling CMOS (Complementary Metal-Oxide-Semiconductor) technology [1,2]. In contrast to other potential materials, such as germanium (Ge) or III–V materials, silicon germanium (SiGe) is considered the most promising channel material for PMOS due to its tunability of band gaps and high hole mobility [3]. However, one of the main challenges in integrating SiGe into the novel devices is obtaining a high-quality interlayer (IL) between high-k gate oxide and SiGe substrate.

To control the interface quality, many methods have been extensively explored, such as plasma (N_2_ or NH_3_) nitridation passivation [4,5], sulfur passivation [6], thermal oxidation [7,8], low-temperature ozone passivation [9,10,11,12] and Si-cap passivation [13]. Among them, low-temperature ozone passivation with low thermal budge and Si-cap passivation with excellent properties of interface are considered the most promising passivation methods. For example, the interface state density (D_it_) of 2.2 × 10^12^ eV^−1^cm^−2^ is attained by using a low-temperature ozone oxidation to passivate the interface of Al_2_O_3_/Si_0.7_Ge_0.3_[11],and the D_it_ of 2 × 10^11^ eV^−1^cm^−2^ for the interface of HfO_2_/Si_0.8_/Ge_0.2_ is realized by using a Si-cap passivation method [14]. However, the technique and mechanism of interface passivation of the HfO_2_/SiGe via low-temperature ozone oxidation or Si-cap method still needs further investigation.

In this paper, we fabricated HfO_2_/IL/Si_0.7_Ge_0.3_ gate stacks MOS capacitors by utilizing low-temperature ozone oxidation and Si cap passivation methods. We carefully compared their electrical properties, and the chemical structure of HfO_2_/IL/SiGe gate stacks. It is found that the post-HfO_2_-depositon (post-O) ozone indirect oxidation is a better choice than a step-by-step procedure (pre-O) method in terms of D_it_ reduction. More importantly, the optimal Si cap method can realize a lower D_it_ of 1.53 × 10^11^ eV^−1^cm^−2^ @ E−E_v_ = 0.36 eV by preventing the formation of Hf-silicate/Hf-germanate and Si oxide originating from the reaction between HfO_2_ and Si_0.7_Ge_0.3_ substrate.

## 2. Materials and Methods

After standard HF-last cleaning, the 30 nm Si_0.7_Ge_0.3_ layer was epitaxially grown in a reduced pressure chemical vapor deposition system (ASM E2000 plus, Amsterdam, The Netherlands) on an 8-inch Si substrate. The low-temperature ozone passivation or Si-cap passivation was employed to passivate the interface of HfO_2_/Si_0.7_Ge_0.3_. For low-temperature ozone passivation samples, the ozone oxidation can occur on the Si_0.7_Ge_0.3_ surface directly (step-by-step procedure (pre-O)) or post HfO_2_ deposition (post-O). The ozone oxidation was carried out in 10% O_3_/O_2_ mixture ambience with the pressure of 3.1 Torr in an atomic-layer-deposition (ALD) chamber (Beneq TFS 200 system, Espoo, Finland). The temperature of the ozone oxidation was 300 °C. For Si-cap passivation, a Si-cap layer was in situ formed on the epitaxial Si_0.7_Ge_0.3_ layer in the same chamber. After the passivation treatment, the W/TiN or W/TiN/HfO_2_ gate stack was deposited as the gate stack of MOS capacitors. Finally, W/TiN/HfO_2_/IL/Si_0.7_Ge_0.3_ MOS capacitors were annealed in the forming gas (10% H_2_, 90% N_2_) at 350 °C for 30 min.

The chemical structures of the HfO_2_/IL/Si_0.7_Ge_0.3_ stacks were studied by X-ray photoelectron spectroscopy (XPS), which was carried out in a Thermo Scientific ESCALAB 250xi (Waltham, MA, USA) system with a photon energy of 1486.7 eV (Al Kα source). The photoelectron emission take-off angle was 90° relative to the sample surface and the pass energy was 15 eV. Moreover, TEM (Transmission Electron Microscope) and EDX (Energy Dispersive X-Ray Spectroscopy) Mapping analysis were performed by using FEI Talos F200X (Hillsboro, MI, USA) to verify the gate stack lattice structure and element content. Multi-frequency capacitance-voltage (C-V) along with conductance-voltage (G-V) measurements were measured using a Keysight 4990 A (Santa Rosa, CA, USA), and leakage-voltage (I-V) was measured using an Agilent B-1500 semiconductor analyzer.

## 3. Results and Discussion

### 3.1. Low-Temperature Ozone Oxidation Passivation of HfO_2_/Si_0.7_Ge_0.3_ Interface

In our previous work, the low-temperature ozone oxidation passivation method has been studied in detail based on Al_2_O_3_/Si_0.7_Ge_0.3_ gate stacks. It was found that oxidation time played an important role to obtain a high-quality interlayer (IL) and should be at least 5 minutes. Otherwise, the unoxidized Ge atoms would be trapped in the IL, causing the IL as well as the relevant electrical properties to deteriorate. Moreover, increasing oxidation time would result in an increase in the ratio of Si^4+^ to Si^3+^ of the oxide interlayer, which can help decrease the D_it_ [15]. Thus, we chose 30 min as the oxidation time, which has proven to be an optimal experimental condition, to passivate the HfO_2_/Si_0.7_Ge_0.3_ interface in this work.

**Figure 1:**
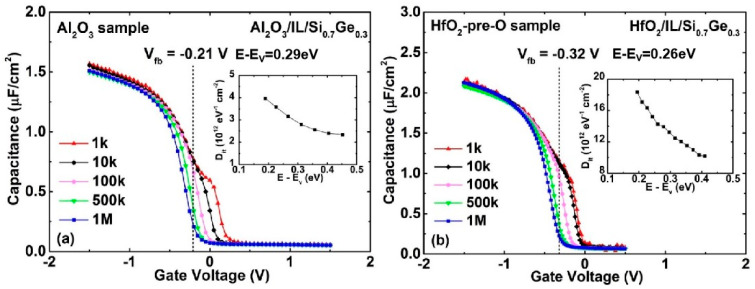
Multi-frequency C-V characteristics of (**a**) Al_2_O_3_ sample (**b**) HfO_2_-pre-O sample with 30 min oxidation time (direct). The insets are their respective energy distributions of D_it_.

Figure 1a,b depicts the multi-frequency (1 kHz to 1 MHz) C-V characteristics of W/TiN/Al_2_O_3_/IL/Si_0.7_Ge_0.3_ (Al_2_O_3_ sample) and W/TiN/HfO_2_/IL/Si_0.7_Ge_0.3_ (HfO_2_-pre-O sample) MOS capacitors treated with 30 min ozone direct oxidation, respectively. The flat band voltages (V_fb_) are also shown in the figures. The frequency dispersion features of the C−V curves observed at gate biases smaller than the V_fb_, are caused by trapping and de-trapping of holes at traps with energies between approximately mid-gap and the Si_0.7_Ge_0.3_ valence band edge, corresponding to the depletion of the Si_0.7_Ge_0.3_ substrate. Comparing Figure 1b with Figure 1a, it is observed that the dispersion feature increases considerably. The energy distributions of the interface state density (D_it_) were extracted using the conductance method [16], and given in their respective inset in Figure 1. We can see that both of the D_it_ of the two samples decreases along with SiGe band gap energy and the maximum D_it_ values appear near the valence band edge (E_v_). However, the maximum value increases from 3.96 × 10^12^ eV^−1^cm^−2^ for the Al_2_O_3_ sample to 2.67 × 10^13^ eV^−1^cm^−2^ for the HfO_2_-pre-O sample. According to our previous work [17], it is known that for 300 °C/30 min ozone oxidation, about 54% of the Ge atoms of the outermost atomic layer of Si_0.7_Ge_0.3_ can be oxidized in the initial stage of oxidation. No more Ge atoms would take part in the oxidation process as the oxidation time increases. The GeO_x_ and SiO_x_ thickness of the formed oxide layer are estimated to be 0.15 nm and 0.72 nm, respectively. Compared with Al_2_O_3_, GeO_x_ is more likely to diffuse into HfO_2_ and cause the HfO_2_/SiGe interface to deteriorate [18]. Therefore, the increased D_it_ of the HfO_2_-pre-O sample can be attributed to tiny amounts of GeO_x_ in the formed oxide layer.

**Figure 2:**
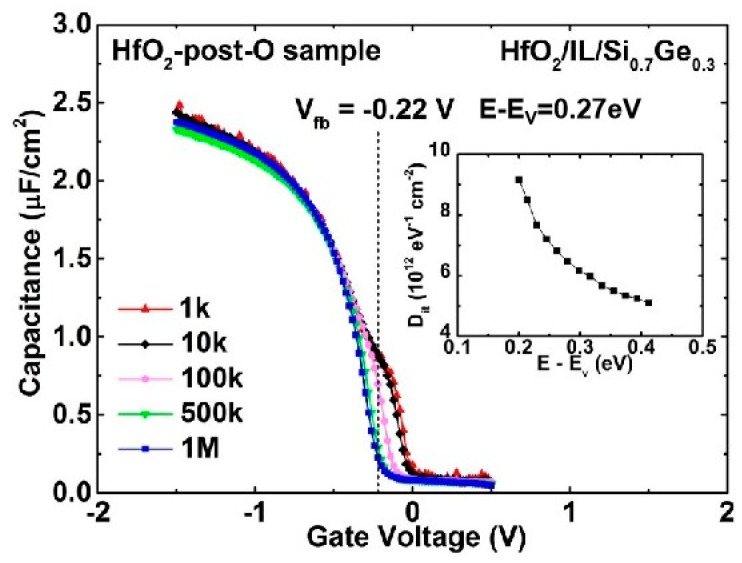
Multi-frequency C-V characteristics of HfO_2_-post-O sample with 30 min oxidation time (indirect). The corresponding energy distributions of D_it_ is given in the inset.

Figure 2 depicts the multi-frequency (1 kHz to 1 MHz) C-V characteristics of W/TiN/HfO_2_/IL/Si_0.7_Ge_0.3_ (HfO_2_-post-O sample) MOS capacitor treated with 30 min ozone indirect oxidation, in which the ozone oxidation was carried out after the deposition of HfO_2_. The corresponding energy distributions of D_it_ is also given in the inset. Compared with Figure 1b, an obvious improvement in the frequency dispersion feature is observed, and the D_it_ value decreases by about 50%. We infer that the improvement may arise from the following two factors. First, due to the barrier effect of the HfO_2_ layer on the diffusion of the oxidizer, the amount of oxygen atoms reaching the interface becomes fewer. Because silicon oxidation is more favorable than germanium oxidation in view of thermodynamic considerations [19], germanium atoms are hardly oxidized in this case. Therefore, almost no GeO_x_ would diffuse into HfO_2_ layer. In addition, the IL thickness of the HfO_2_-post-O sample is smaller than that of the HfO_2_-pre-O sample, which means the amounts of the Ge atoms accumulating at the IL/SiGe interface decrease accordingly. The experimental results prove that the post-O method is a promising technology to realize an HfO_2_/IL/SiGe gate stack with small D_it_.

The Al_2_O_3_ sample, HfO_2_-pre-O sample and HfO_2_-post-O sample were compared on capacitance equivalent oxide thickness (CET) at −1.5 V bias voltage in accumulation. The CETs of each are 2.28 nm, 1.5 nm and 1.37 nm respectively. Comparing the Al_2_O_3_ sample with the HfO_2_, the CET of the Al_2_O_3_ sample is bigger. The HfO_2_-post-O sample decreased the CET, compared to the HfO_2_-pre-O sample. This is supposed to be related to the diffusion of GeO_x_. In general, the diffusion of GeO_x_ is less in Al_2_O_3_ and HfO_2_-post-O. Using the post-O method can limit the diffusion of GeO_x_ in HfO_2_. The diffusion of GeO_x_ affects not only the CET but also the leakage current.

**Figure 3:**
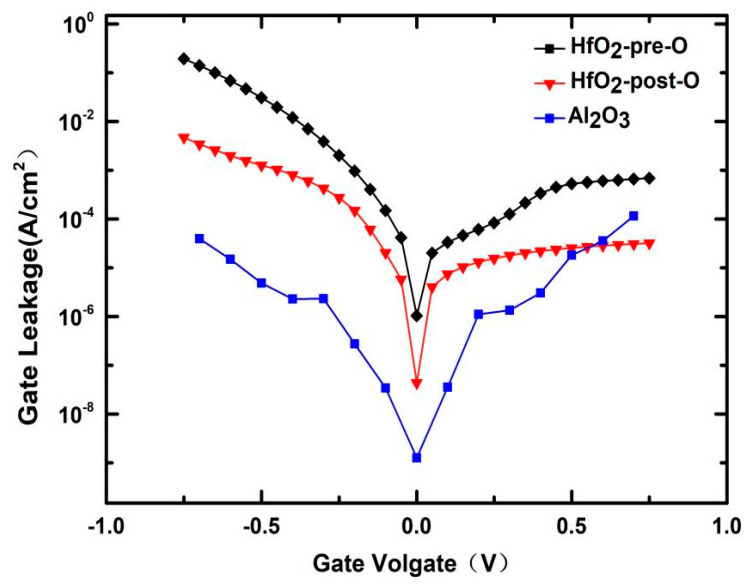
Gate leakage of Al_2_O_3_ sample, HfO_2_-pre-O sample and HfO_2_-post-O sample.

Figure 3 shows the gate Leakage of the Al_2_O_3_ sample, HfO_2_-pre-O sample and HfO_2_-post-O sample. Because GeO_x_ is not easily diffused in Al_2_O_3_, the leakage current is minimal for the Al_2_O_3_ sample. Comparing with the HfO_2_-pre-O sample, the leakage current HfO_2_-post-O sample can be reduced by an order of magnitude.

### 3.2. Si-Cap Passivation of HfO_2_/Si_0.7_Ge_0.3_ Interface

**Figure 4:**
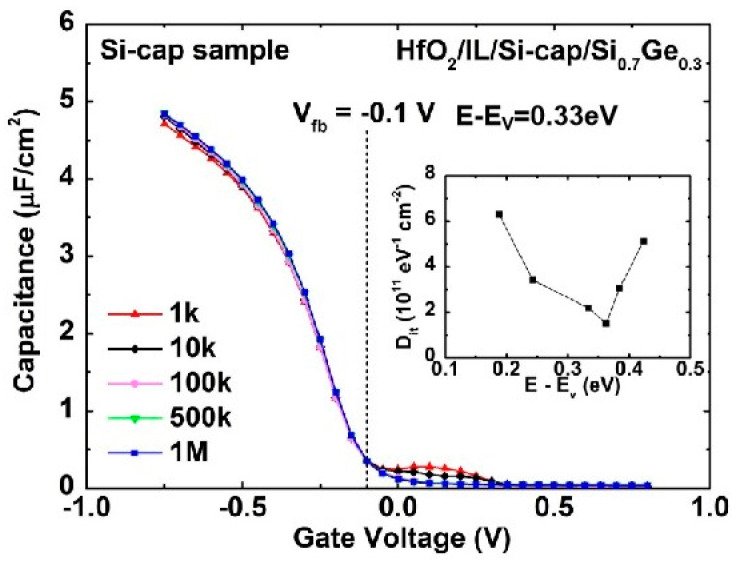
Multi-frequency C-V characteristic of W/TiN/HfO_2_/IL/Si-cap/Si_0.7_Ge_0.3_ MOS capacitor with 1 nm Si-cap.

**Figure 5:**
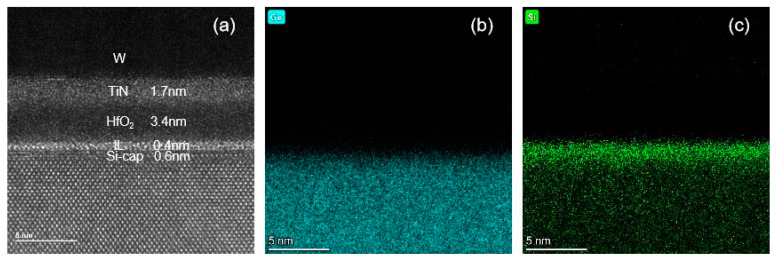
(**a**) HRTEM, (**b**) Ge, and (**c**) Si element EDX mapping of the W/TiN/HfO_2_/IL/Si-cap/Si_0.7_Ge_0.3_ MOS capacitor with 1 nm Si-cap.

To further reduce the D_it_ of the HfO_2_/Si_0.7_Ge_0.3_ interface, Si-cap passivation is in situ performed on the Si_0.7_Ge_0.3_ layer with different thicknesses. It is found that if the Si cap thickness is larger than or equal to 2 nm, there is a step observed in its C-V curve because a second channel is formed in the Si cap layer. This can be avoided by further thinning of the Si cap layer to 1 nm. Moreover, multi-frequency C-V curves (1 kHz to 1 MHz) of the W/TiN/HfO_2_/IL/Si-cap/Si_0.7_Ge_0.3_ MOS capacitor with 1 nm Si-cap are measured and shown in Figure 3. It is worthy to note that the frequency dispersive feature is obviously improved compared with the above ozone passivation. However, the CET of the Si-cap sample from Figure 4 may be inaccurate due to the large gate leakage in the accumulation region. In addition, it can be seen that the carriers are mainly confined in the Si_0.7_Ge_0.3_ layer under this optimal Si-cap thickness due to its large valance band offset. For quantitative analysis, the D_it_ of 1.53 × 10^11^ eV^−1^cm^−2^ @ E−E_v_ = 0.36 eV is attained by using the conductance method. Meanwhile, HRTEM, Si and Ge element EDX mapping of the W/TiN/HfO_2_/IL/Si-cap/Si_0.7_Ge_0.3_ MOS capacitor with 1nm Si-cap is also implemented and shown in Figure 5. It is found that there is a ~0.6 nm Si capping on the Si_0.7_Ge_0.3_ with a smooth and high-quality interfacial layer. The reduction of Si cap thickness of 0.4 nm is due to the oxidation of Si cap layer in the process of MOS capacitor fabrication. Therefore, 1-nm Si-cap in situ epitaxial grown is chosen as the optimal Si-cap thickness.

**Figure 6:**
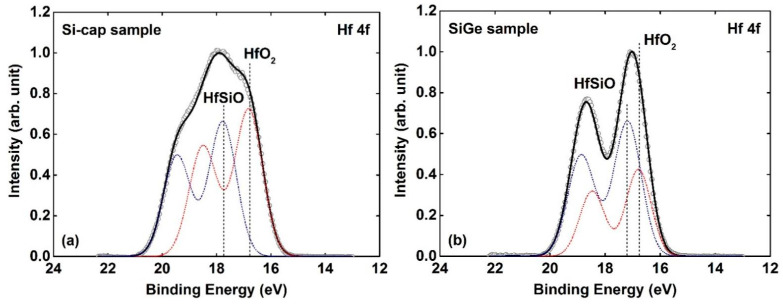
The fitted Hf 4f core-level spectra of (**a**) Si-cap sample and (**b**) SiGe sample. The blue and red dot lines denote the Hf 4f photoelectron from Hf-O-Si and/or Hf-O-Ge bonds and Hf-O bonds in HfO_2_, respectively.

For the purpose of investigating the chemical structure of the HfO_2_/IL/Si-cap/Si_0.7_Ge_0.3_ gate stack (Si-cap sample), X-ray photoelectron spectroscopy (XPS) technology is implemented. The chemical structure of the HfO_2_/Si_0.7_Ge_0.3_ gate stack (SiGe sample), in which HfO_2_ is deposited on Si_0.7_Ge_0.3_ directly, is also analyzed as a control sample. Gaussian-Lorentzian line shapes are used for deconvolution of all the spectra after standard Shirley background subtraction [20]. Figure 6a,b shows the Hf 4f core-level spectra of the Si-cap sample and SiGe sample, respectively. The spectra are both fitted with two component peaks. For the Si-cap sample (shown in Figure 6a), the Hf 4f spectrum consists of a main component at 16.8 eV related to the Hf-O bands in HfO_2_, and a second component shifted by ~0.9 eV to higher binding energy, which is from the Hf-O-Si and/or Hf-O-Ge bonds. Because the electro-negativities of the Hf second neighbors (i.e., Si and Ge) are similar, it is difficult to distinguish the two contributions of Hf-O-Si and Hf-O-Ge bonds by XPS. It is worth noting that for the SiGe sample (shown in Figure 6b), the areal intensity of Hf-O-Si/Hf-O-Ge is much more than that of Hf-O. This suggests that a large portion of HfO_2_ would react with SiGe to form Hf-silicate/Hf-germanate during the HfO_2_ ALD deposition process. In addition, no feature of lower banding energy (14.3 eV–14.8 eV) is detected, indicating that no metallic Hf-Si and/or Hf-Ge are formed in the two samples.

**Figure 7:**
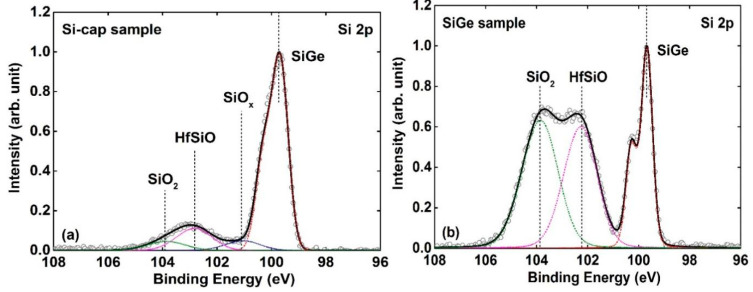
The fitted Si 2p core-level spectra of (**a**) Si-cap sample (**b**) SiGe sample. The red, blue, magenta, and green dot lines denote the Si 2p of SiGe, SiO_x_, HfSiO, and SiO_2_, respectively.

**Figure 8:**
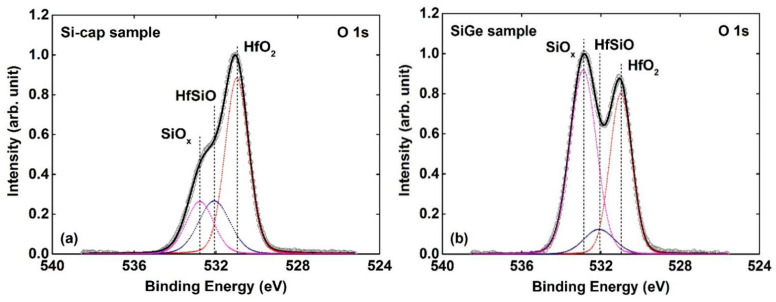
The fitted O 1s core-level spectra of (**a**) Si-cap sample (**b**) SiGe sample. The red, blue, and magenta dot lines denote the O1s photoelectron from HfO_2_, HfSiO, and SiO_x_, respectively.

Figure 7a,b shows the Si 2p core-level spectra of the Si-cap sample and the SiGe sample, respectively. The spectra are decomposed into four component peaks i.e., Si 2p photoelectron from SiGe (~99.7 eV), SiO_x_ (~101.2 eV), HfSiO (~102.8 eV), and SiO_2_ (~103.9 eV). For the Si-cap sample (shown in Figure 7a), the high-binding energy shoulder (101 eV~105 eV) contains few amounts of Si oxide (SiO_x_ and SiO_2_) and Hf-silicate (HfSiO). When compared with the Si-cap sample, an obvious increase in the areal intensity of the high-binding energy shoulder (101 eV~105 eV) can be observed for the SiGe sample, and there is no peak corresponding SiO_x_. Figure 8a,b shows the O 1s core-level spectra of the SiGe sample and Si-cap sample, respectively. The spectra are fitted by the O 1s of SiO_x_ (~532.8 eV), HfSiO (~532.08 eV) and HfO_2_ (~531 eV). We can see that the O 1s photoelectron mainly originates from HfO_2_ for the Si-cap sample, while that of the SiGe sample is mainly from SiO_x_ and HfSiO. This is consistent with the previous discussions about Hf 4f and Si 2p spectra. All of these results indicate that the interfacial region of the HfO_2_/SiGe (SiGe sample) is a composite of large amounts of HfSiO (and/or HfGeO) and Si oxide (SiO_2_). In other words, Si-cap can prevent the formation of Hf-silicate/Hf-germanate and Si oxide originating from the reaction between HfO_2_ and SiGe substrate, and obtain an excellent HfO_2_/SiGe interface.

## 4. Conclusions

In summary, the interface passivation of the HfO_2_/Si_0.7_Ge_0.3_ stack is systematically investigated based on low-temperature ozone oxidation and Si-cap methods. Compared with pre-O method, the D_it_ of the post-O sample decreases by about 50% due to less GeO_x_ available in the IL layer. However, the D_it_ of the HfO_2_/IL/Si_0.7_Ge_0.3_ gate stack still has room to be further optimized. Finally, an excellent HfO_2_/Si_0.7_Ge_0.3_ interface with a D_it_ of 1.53 × 10^11^ eV^−1^cm^−2^ @ E−E_v_ = 0.36 eV is attained under the optimal Si cap method by preventing the formation of Hf-silicate/Hf-germanate and Si oxide from the reaction HfO_2_ and Si_0.7_Ge_0.3_ substrate.

## Data Availability

Not applicable.
